# Author Correction: Unsupervised classification of brain-wide axons reveals the presubiculum neuronal projection blueprint

**DOI:** 10.1038/s41467-024-53487-9

**Published:** 2024-10-28

**Authors:** Diek W. Wheeler, Shaina Banduri, Sruthi Sankararaman, Samhita Vinay, Giorgio A. Ascoli

**Affiliations:** Center for Neural Informatics, Krasnow Institute for Advanced Studies and Bioengineering Department, College of Engineering & Computing, George Mason University, Fairfax, VA USA

**Keywords:** Neural circuits, Classification and taxonomy

Correction to: *Nature Communications* 10.1038/s41467-024-45741-x, published online 20 February 2024

The original version of this Article omitted a statement acknowledging that digital object identifiers were available in the Source Data Supplement file for the Janelia MouseLight dataset.

The following sentence should be added to the end of the Data Availability statement.

“The Janelia MouseLight reconstructions from layer 6 of the primary motor cortex and the presubiculum are available for citation by way of their individual digital object identifiers, as provided in the Source Data Supplement file.”

The original version of this Article also omitted the digital object identifier Source Data Supplement file for the Janelia MouseLight dataset.

This has been corrected in the PDF and HTML version of the article.

